# Outcomes after Total Hip Arthroplasty Using a Cementless S‐ROM Modular Stem for Patients with High Hip Dislocation Secondary to Hip Pyogenic Arthritis

**DOI:** 10.1111/os.12485

**Published:** 2019-06-26

**Authors:** Yang Yang, Qiu‐Ping Yu, Shao‐Lin Wang, Sheng‐Li Zhang, Juan Li, Yi Zhou, Hua‐Quan Fan, Xin Zhang, Yu Zhou, Min Zhou, Ming‐Quan Zhou, Ning Li, Jun‐Li Liu

**Affiliations:** ^1^ Department of Integrated Traditional and Western Medicine West China Hospital, Sichuan University Chengdu China; ^2^ Center for Joint Surgery, Southwest Hospital, Third Military Medical University (Army Medical University) Chongqing China; ^3^ Department of Orthopaedics Chongqing General Hospital Chongqing China

**Keywords:** High dislocation, Pyogenic arthritis, S‐ROM stem, Subtrochanteric shortening osteotomy, Total hip arthroplasty

## Abstract

**Objective:**

To evaluate the midterm results of the cementless S‐ROM modular femoral stem used with subtrochanteric transverse shortening osteotomy for the treatment of high hip dislocation secondary to hip pyogenic arthritis.

**Methods:**

We retrospectively reviewed the data of 49 patients (49 hips) with an average infection quiescent period of 37.4 years who underwent cementless total hip arthroplasty (THA) with simultaneous subtrochanteric transverse shortening osteotomy from July 2008 to June 2012. There were 23 men and 26 women with a mean age of 44.3 years at the time of surgery. The following clinical outcomes were evaluated: the Western Ontario and McMaster Universities Arthritis Index (WOMAC) score, Harris hip score (HSS), modified Merle d'Aubigne‐Postel hip (MAP) score, low back pain visual analog scale score, 12‐item short‐form health survey questionnaire score, limp, and Trendelenburg sign. Radiographic outcomes and complications were also evaluated.

**Results:**

The mean follow‐up period was 8.7 years (range, 5.5–10 years). No infection recurrence was observed after THA. The average HSS significantly improved from 45.0 to 84.8. The WOMAC score improved from 70.1 ± 3.5 (range, 65–76) to 43.1 ± 13.4 (range, 21–67). The modified MAP score improved from 5.9 ± 1.9 (range, 3–9) to 14.3 ± 2.4 (range, 11–18). The low back pain visual analog scale score, 12‐item short‐form health survey questionnaire score, limp, and Trendelenburg sign also improved significantly. The average limb length discrepancy decreased from 39.6 mm (range, 30–55 mm) to 7.2 mm (range, 0–22 mm). Two patients had temporary sciatic nerve paralysis but recovered within 6 months without any functional defects; one had an intraoperative fracture fixed by cerclage wires. One hip required revision surgery because of femoral stem aseptic loosening.

**Conclusions:**

The cementless S‐ROM modular femoral stem used with subtrochanteric transverse shortening osteotomy is safe and effective for high hip dislocation secondary to pyogenic arthritis and provides satisfactory midterm results. Significant improvements in clinical function were observed, as were high rates of stable fixation of the cementless implant, restoration of more normal limb lengths, and a low incidence of complications.

## Introduction

Total hip arthroplasty (THA) has been considered the most successful method for treating high hip dislocation^1^, ^2^. However, it is one of the most technically challenging procedures for patients with high hip dislocation secondary to pyogenic arthritis because of the potential risk of recurrent infection and distinctive anatomic abnormalities such as leg length discrepancy, dysplasia of the acetabulum and femur, deficient bone stock, severe flexion deformities, joint stiffness, and contracture of soft tissue that can alter the locations of the profunda femoris artery and femoral nerve, artery, and vein^3^. The main concerns regarding THA for high hip dislocation are the dysplastic acetabulum and femur, restoration of the anatomical rotation center of the hip, and leg length. The dysplastic acetabulum may require a small acetabular component and/or structural bone grafting. Increased anterior bowing of the proximal femur and narrowness of the femoral canal with metaphyseal/diaphyseal mismatch cause difficulties with canal preparation^3^. Femoral reduction may be difficult and is associated with a high risk of neurologic injury because of the high dislocation and soft tissue contracture. Furthermore, higher complication rates were reported for THA for high hip dislocation; these complications included intraoperative fractures, recurrent infection, dislocation, osteolysis, mechanical loosening, and revisions^4^, ^5^, ^6^, ^7^.

Distinctive anatomic abnormalities in high hip dislocation are ideal indications for subtrochanteric shortening osteotomy and a modular femoral stem^2^, ^8^. Subtrochanteric shortening osteotomy allows simultaneous shortening of the femur, preservation of the proximal femoral metaphysic, and correction of the rotational abnormalities^9^. Transverse, oblique, double‐chevron, step‐cut, and V‐shaped subtrochanteric shortening osteotomy procedures have been reported in the published literature^10^, ^11^. Modularity optimizes proximal and distal implant stability while permitting free adjustments to the leg length, anteversion, and offset to provide physiological kinematics reconstruction of the hip. The torsional stability of the fluted modular stem makes it a good choice for subtrochanteric osteotomy because of the resulting stabilization and reduction of high dislocation of the hip^8^, ^12^.

We hypothesized that the cementless S‐ROM modular femoral stem used with subtrochanteric transverse shortening osteotomy would improve the outcomes of THA for patients with high hip dislocation secondary to pyogenic arthritis. The aims of the present study were: (i) to determine the efficiency of this strategy for THA for patients with high hip dislocation secondary to pyogenic arthritis; (ii) to evaluate the functional outcomes of patients; and (iii) to evaluate patient complications.

## Methods

### *Inclusion and Exclusion Criteria*

After approval from the ethics committee of our institute, this retrospective study was conducted. Patients were recruited who underwent unilateral THA from July 2008 to June 2012 in our hospital.

Inclusion criteria were: (i) age older than 18 years; (ii)high hip dislocation (Crowe type IV according to Crowe's classification^13^) secondary to pyogenic arthritis; (iii) an infection quiescent period >10 years; and (iv) THA using a cementless S‐ROM modular femoral stem with subtrochanteric transverse shortening osteotomy. Patients were excluded based on the following criteria: (i) recent pyogenic arthritis of the hip; (ii) mild‐to‐moderate hip dislocation; (iii) THA without S‐ROM modular femoral stem; and (iv) THA without subtrochanteric shortening osteotomy or with oblique, double‐chevron, step‐cut, and V‐shaped subtrochanteric shortening osteotomy. Informed consent was obtained from all patients included in this study.

### *Patient Information*

A total of 52 patients with unilateral high dislocations of the hip secondary to pyogenic arthritis who underwent primary cementless THA (S‐ROM stem) combined with simultaneous subtrochanteric transverse shortening osteotomy were enrolled for review. Severe pain and functional impairment while performing daily activities were indications for THA. Three patients were lost to follow‐up after surgery. Therefore, 49 patients (23 men and 26 women) with a mean age of 44.3 years (range, 23–63 years) at the time of surgery were investigated. Preoperative clinical and radiographic evaluations were performed for all patients. The mean body mass index was 23.4 kg/m^2^ (range, 18.7–32.7 kg/m^2^). Demographic data of patients are presented in Table 1. The average preoperative limb length discrepancy (LLD) was 39.6 mm (range, 30–50 mm). All patients had positive Trendelenburg test results. Hip joint stiffness existed in all patients, and the flexion range of motion (ROM) was 75° (range, 0°–130°). The minimum follow‐up period was 5.5 years (mean, 8.7 years; range, 5.5–10 years).

**Table 1:** Demographics of patients

Parameters	Values
Gender (male/female)	23/26
Age (years)	44.3 ± 6.6 (range, 23–63)
Weight (kg)	58.9 ± 8.5 (range, 41–80)
Height (cm)	162.7 ± 7.7 (range, 150–176)
Body mass index (kg/m^2^)	22.4 ± 3.6 (range, 15.4–28.9)

The average quiescent period of hip pyogenic arthritis was 37.4 years (range, 17–62 years). White blood cell counts, C‐reactive protein levels, and erythrocyte sedimentation rates were examined preoperatively. Frozen‐section analysis was used to detect infections intraoperatively. Synovial fluid and excised specimens were collected for bacterial cultures. Active infection was identified when at least two of the following three characteristics were present: grossly infected tissues observed at the time of surgery; final histopathology with an average of more than five polymorphonuclear leukocytes per high‐power field; and growth of bacteria on solid media on at least two culture specimens^14^. During surgery, norvancomycin 0.4 g was administered intravenously and used continuously for 2 days (0.4 g twice daily).

### *Surgical Technique*

All patients underwent surgery in the lateral decubitus position using the posterolateral approach to the hip under general or spinal anesthesia. Cementless femoral and acetabular components were used for all surgeries. The acetabular components were placed medial to the wall in the original acetabulum in all hips. The acetabulum was reamed gradually to reach the medial wall of the true acetabulum, starting with very small reamers. Small‐diameter acetabular implants were often needed for the shallow and small acetabulum to achieve sufficient coverage. The median diameter of the acetabular cup was 46 mm (range, 40–52 mm). A structural femoral head autograft was performed in 8 hips to provide adequate superolateral coverage of the cup if the acetabular bone was deficient. A Pinnacle (DePuy, Warsaw, IN, USA) porous‐coated acetabular component was inserted in the anatomic acetabular position using the press‐fit technique, and dome screws were used for acetabular cup fixation. A ceramic liner or ultrahigh‐molecular‐weight polyethylene was coupled with the femoral head. Ceramic‐on‐ceramic wear‐bearing material was used in 23 hips, and poly‐on‐metal was used in 26 hips.

Transverse subtrochanteric femoral shortening resection with a length of 30 to 55 mm (38.5 ± 6.3 mm) was required to restore the anatomical rotation center of the hip for all patients. This was performed by resecting the femur below the lesser trochanter, usually at 1–2 cm beneath the lesser trochanter, in accordance with the preoperative template and intraoperative examination. Longitudinally split fragments from the resected cylindric bone of the femur were placed around the osteotomy site as a structural allograft, fixed with cerclage wires, and augmented by morselized cancellous bone autografts from the resected femoral heads to enhance rotational stability and accelerate bone union of the osteotomy site^2^. The rotational alignment of the two fragments was adjusted to allow approximately 15°–20° of anteversion of the femoral stem^15^, ^16^. A cementless S‐ROM (DePuy) femoral stem with a cobalt–chrome alloy or ceramic head (range, 22–36 mm) was inserted after the distal part of the femur was prepared for implantation. To reduce the risk of intraoperative fractures, prophylactic cerclage wires were used before femoral stem insertion.

All patients were encouraged to perform early limb exercises immediately after surgery. The mean length of the postoperative hospital stay was 5.4 days (range, 3–10 days). All patients were allowed to stand within 2 days, and they walked with partial weight‐bearing for the first 6 weeks. Then, gradually progressive full weight‐bearing was allowed depending on the stability of the femoral stem and positive osseous healing at the osteotomy site.

### *Outcome Measures*

#### Clinical Evaluations

All patients were evaluated at similar intervals. Clinical evaluations were performed using the Western Ontario and McMaster Universities Arthritis Index (WOMAC)^17^, the modified Merle d'Aubigne‐Postel (MAP) hip score, the Harris hip score (HSS)^18^, the low back pain visual analog scale (VAS) score, and the 12‐item short‐form health survey questionnaire (SF‐12)^19^ preoperatively and at regular postoperative intervals (immediately after surgery, at 3 months and 6 months postoperatively, at 1 year postoperatively, and then once yearly thereafter)^2^. Preoperative and postoperative Trendelenburg signs, limping, and complications such as intraoperative femoral fractures, recurrent infection, dislocation, neurologic injury (temporary or permanent), and deep venous thrombosis were also recorded.

#### Radiographic Evaluations

Anteroposterior and lateral radiographs of the hip and a full‐length view of the lower extremities were obtained and reviewed by the same observer at each follow‐up time point. Leg lengthening was defined as the distance between the top of the greater trochanter preoperatively and postoperatively observed on radiographs minus the amount of intraoperative femoral resection^20^. The cup inclination was measured as previously described by Mu *et al*.^4^ Bone union at the osteotomy site was assessed on postoperative radiographs according to the method of Masonis *et al*.^21^ Osteointegration of the femoral prosthesis was classified as bone ingrowth, stable fibrous ingrowth, or unstable^22^. Radiolucent lines and osteolytic lesions around the femoral component (according to the method of Gruen *et al*.^23^) and the acetabular component (according to the method of DeLee and Charnley^24^) were analyzed. Femoral component loosening was evaluated using a radiographic analysis, as described by Engh *et al*.^25^ In addition, acetabular component loosening was diagnosed when there was a change in the position of the component or a continuous radiolucent line that was more than 2 mm wide around the component^26^. Subsidence of the femoral component was evaluated according to the method of Heekin *et al*.^27^ Heterotopic ossification was evaluated using the method of Brooker *et al*.^28^

### *Statistical Analysis*

Categorical variances are presented as frequencies, and continuous variances are presented as means and ranges. The χ^2^‐test was used to compare preoperative and postoperative categorical variance results. A two‐sided Student's paired *t*‐test was used to compare preoperative and postoperative continuous variance results. Significance was determined as *P* < 0.05. Statistical analysis was performed using SPSS 13.0 (SPSS, Chicago, IL, US).

## Results

### *Follow‐up*

All patients were evaluated at similar intervals (immediately after surgery, at 3 months and 6 months postoperatively, at 1 year postoperatively, and then once yearly thereafter). The mean follow‐up period was 8.7 years (range, 5.5–10 years).

### *General Results*

All patients had preoperative white blood cell counts, C‐reactive protein levels, and erythrocyte sedimentation rates that were within the reference ranges. All intraoperative examinations were negative for infection. Intraoperatively frozen sections, synovial fluid, and excised specimen cultures did not show any evidence of infection. Infection recurrence was not observed after THA in this series.

### *Functional Evaluation*

The mean WOMAC score, modified MAP score, HHS, low back pain VAS score, SF‐12 score, limp, and Trendelenburg sign significantly improved postoperatively compared with preoperatively (Table 2).

**Table 2:** Clinical parameters preoperatively and at the final follow‐up

Indices	Preoperative	Postoperative	*P‐*value
WOMAC score (mean ± SD) (point)	70.1 ± 3.5 (range, 65–76)	43.1 ± 13.4 (range, 21–67)	<0.001*
Modified MAP			
Mean in points (mean ± SD)	5.9 ± 1.9 (range, 3–9)	14.3 ± 2.4 (range, 11–18)	<0.001*
Pain	2.6 ± 1.1 (range, 1–4)	5.0 ± 0.9 (range, 4–6)	<0.001*
Walking	1.9 ± 0.9 (range, 1–3)	5.0 ± 0.8 (range, 4–6)	<0.001*
ROM	2.0 ± 0.8 (range, 1–3)	4.8 ± 0.8 (range, 4–6)	<0.001*
Harris hip score (mean ± SD)			
Mean in points	45.0 ± 10.6 (range, 30–63)	84.8 ± 6.6 (range, 75–95)	<0.001*
Rating (number of hips)			
Excellent (90–100 points)	0	15	
Good (80–90 points)	0	22	
Fair (70–79 points)	0	12	
Poor (<70 points)	49	0	
Low back pain VAS score (number of hips)			
None (0 point)	40	46	
Mild (1–3 points)	5	3	
Moderate (4–6 points)	3	0	
Severe (7–10 points)	1	0	
Length discrepancy of limbs			
Mean in mm (mean ± SD) (mm)	39.6 ± 5.4	7.2 ± 4.3	<0.001*
<10 mm (number of hips)	0	36	
10–20 mm (number of hips)	0	12	
21–30 mm (number of hips)	0	1	
31–40 mm (number of hips)	26	0	
41–50 mm (number of hips)	22	0	
>50 mm (number of hips)	1	0	
Limp (severe/moderate/mild/none, *n*)	29/12/8/0	0/2/7/40	<0.001*
SF‐12			
PCS	9.3 ± 3.5 (range, 5–15)	20.1 ± 2.4 (range, 16–24)	<0.001*
MCS	13.3 ± 2.8 (range, 9–18)	23.6 ± 2.7 (range, 20–29)	<0.001*

MAP, Merle d'Aubigne and Postel; MCS, mental component summary; PCS, physical component summary; ROM, range of motion; SF‐12, 12‐item short‐form health survey questionnaire; VAS, visual analogue scale; WOMAC, Western Ontario and McMaster Universities Arthritis Index.

* Statistically significant (*P* < 0.05).

### *Radiographic Evaluation*

All hips that underwent subtrochanteric femoral shortening resection had healed by the time of the follow‐up visit at 1 year without any complications. The femoral head was reduced into the true acetabulum for each hip. The mean LLD decreased from 39.6 mm (range, 30–55 mm) preoperatively to 7.2 mm (range, 0–22 mm) postoperatively. Four patients reported longer limb lengths on the operative side; however, the physical measurements indicated that no limb on the operative side was longer than the limb on the contralateral side. This feeling of LLD dissipated by the time of the 6‐month follow‐up visit.

**Figure 1:**
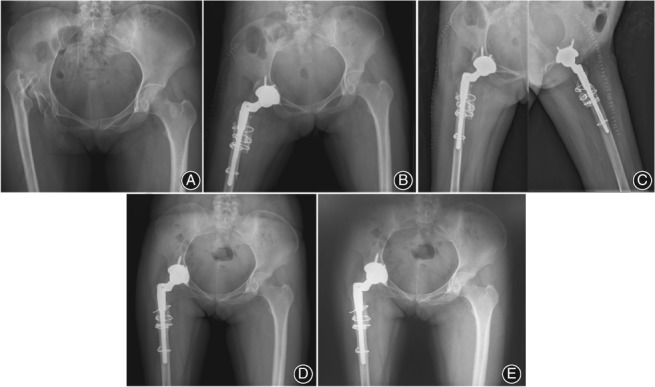
A 28‐year‐old woman with Crowe type IV high hip dislocation secondary to pyogenic arthritis who had a quiescent period of 25 years between infection and right total hip arthroscopy (THA). (A) Preoperative anteroposterior (AP) pelvic radiograph. (B) Postoperative AP pelvic radiograph immediately after right THA. (C) Postoperative AP and oblique hip radiographs immediately after right THA. (D) Postoperative AP pelvic radiograph 5 years after right THA. (E) Postoperative AP and oblique hip radiographs 8 years after right THA.

**Figure 2:**
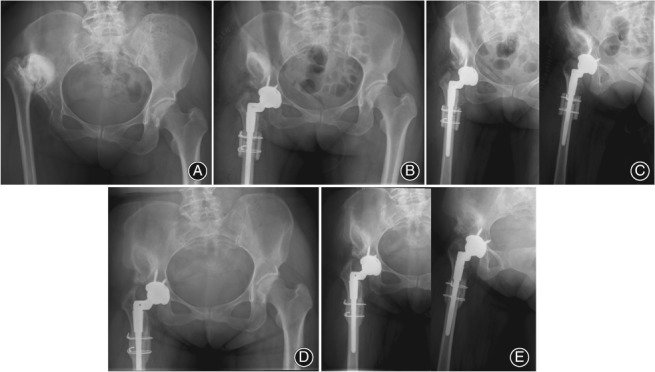
A 42‐year‐old woman with Crowe type IV high hip dislocation secondary to pyogenic arthritis who had a quiescent period of 20 years between infection and right total hip arthroscopy (THA). (A) Preoperative anteroposterior (AP) pelvic radiograph. (B) Postoperative AP pelvic radiograph immediately after right THA. (C) Postoperative AP and oblique hip radiographs immediately after right THA. (D) Postoperative AP pelvic radiograph 7 years after right THA. (E) Postoperative AP and oblique hip radiographs 7 years after right THA.

**Figure 3:**
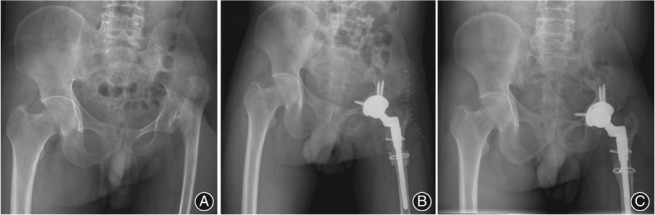
A 44‐year‐old man with Crowe type IV high hip dislocation secondary to pyogenic arthritis who had a quiescent period of 27 years between infection and right total hip arthroscopy (THA). (A) Preoperative anteroposterior (AP) pelvic radiograph. (B) Postoperative oblique hip radiographs immediately after right THA. (C) Postoperative AP pelvic radiograph 8 years after right THA.

All acetabular components remained *in situ* and stable according to radiographs at the time of the last follow‐up, and structural femoral head autografts showed excellent incorporation into the host bone (Figs 1, 2, 3). Forty‐seven hips were identified as stable bone ingrowth, and two hips were identified as stable fibrous ingrowth at the final follow‐up according to the Engh classification. A radiolucent line smaller than 1 mm in zone 1 of the acetabulum was identified in 2 patients. Small focal osteolysis was noted in zones 1 and 2 of the acetabulum in 1 patient; this required no revision at the time of the last follow‐up. Small focal osteolysis located in zone 1 and/or zone 7 was identified in the femur in 3 patients. No hip had distal osteolysis. One hip had stem subsidence and subsequent loosening of the femoral component 7 years postoperatively; stem revision was performed, and the new femoral component was stable. Asymptomatic heterotopic ossification was seen in three hips (two were class I and one was class II according to the Brooker classification system^28^) on follow‐up radiographs.

### *Complications*

One hip had intraoperative femoral fractures that were fixed by cerclage wires; these had healed by the time of the follow‐up visit at 1 year without further sequelae. One patient experienced postoperative dislocation (7 days after surgery) and was treated with closed reduction; the dislocation did not recur. No evidence of deep venous thrombosis was found in any patient. Two patients experienced temporary sciatic nerve paralysis postoperatively, but they recovered within 6 months without any functional defects.

## Discussion

Total hip arthroplasty is challenging when patients have high dislocation of the hip secondary to pyogenic arthritis. Severe anatomic deformities, soft tissue contractures, and the potential risk of recurrent infection complicate this surgery. Previous infection and repeated surgeries may result in severe soft tissue contractures, altered locations of the femoral nerve, artery, and vein, and altered directions of the abductor muscles. Furthermore, the narrowness of the femoral intramedullary canal and increased anterior bowing of the proximal femur make canal preparation difficult. Therefore, it is difficult to achieve reduction of the femoral head into the true acetabulum and restore the abductor function without neurovascular injury.

Yan *et al*. reported a reduction technique for severe developmental high dislocation without femoral shortening osteotomy using an intravenous injection of rocuronium combined with continuous strong traction that resulted in satisfactory outcomes^29^. However, we think it is more appropriate to use femoral shortening osteotomy for high dislocation of the hip secondary to pyogenic arthritis because there are more severe soft tissue contractures and neurovascular anomalies involved. It was reported that the prevalence of nerve palsy for those who underwent THA was between 5.5% and 16% for patients who had childhood pyogenic arthritis^3^, ^30^, ^31^, ^32^, which was higher than that reported for patients with Crowe type IV developmental dysplasia of the hip (5%–11.3%)^4^, ^9^, ^20^, ^33^. In this series, 2 patients (4.1%) experienced temporary sciatic nerve paralysis postoperatively and recovered within 6 months without any functional defects. These 2 patients had severe soft tissue contracture and required extensive soft tissue release.

Many osteotomy techniques have been reported, such as the oblique, transverse, step‐cut, V‐shaped, and double‐chevron techniques^34^. In this series, we used transverse subtrochanteric shortening osteotomy. Compared with nonosteotomy techniques, subtrochanteric shortening osteotomy can simplify reduction and ensure correction of the femoral deformity, but it may lead to complications such as dislocation, loosening, osteotomy site nonunion, and instability^33^. Transverse osteotomy, which has a limited bony contact area and lacks inherent rotational stability, is especially vulnerable to these possible complications^34^, ^35^. To address these concerns, the longitudinally split fragments from the resected cylindric femur bone were placed around the osteotomy site as a structural allograft, fixed with cerclage wires, and augmented by morselized cancellous bone autografts from the resected femoral heads to accelerate bone union of the osteotomy site and enhance rotational stability^34^. In this series, all hips that had undergone transverse subtrochanteric femoral shortening osteotomy had healed by the time of the follow‐up visit at 1 year, and no nonunion or prostheses loosening was found. All patients experienced successful autograft bone graft fusion. Therefore, transverse subtrochanteric shortening osteotomy can result in good healing and sufficient primary stability. The S‐ROM stem is a cementless assembled cylindrical prosthesis. Its stem was designed to enable free adjustment of the anteversion angle with theoretic maximal proximal and distal fill^36^; therefore, it is helpful for restoring the appropriate anteversion. Its proximal sleeve can offer maximal contact with the host bone, thereby providing favorable rotational stability. The straight stem with a distal fluted design could potentially offer additional rotational stability. Proximal stability and distal rotational stability are indispensable when attempting to achieve bony union without failure after osteotomy. Furthermore, the porous‐coated metaphyseal sleeves of the S‐ROM stem for bone ingrowth are designed to convert hoop and shear stresses to compressive forces at the sleeve–bone interface, which will seal the medullary cavity from wear debris and reduce the stress shielding effect. In this series, one femoral stem was loose at 7 years after THA; therefore, revision surgery was performed, and the new femoral component was stable.

It has been recommended that there should be a quiescent period of infection more than 10 years before THA is performed to avoid the potential risk of recurrent infection^37^, ^38^, ^39^. In addition, histologic and bacteriologic sampling should be thorough and extensive to maximize the chances of identifying residual bacteria^38^, ^39^. In this series, the average quiescent period of infection was 37.4 years (range, 17–62 years). Hematological parameters, frozen sections, synovial fluid, and excised specimen cultures did not yield any evidence of bacterial infection in any patients, including the patient who underwent revision surgery 7 years after THA. No recurrent infection was reported in this series. Pain and joint function improved considerably after surgery according to the WOMAC score, the modified MAP score, and the HSS. Limp, low back pain, LLD, and health‐related quality of life also improved significantly.

The incidence of complications was low in this series. One patient (2%) underwent revision until the final follow‐up examination because of femoral stem aseptic loosening; however, this rate was lower than that reported by Kim *et al*. (revision rate of 5% after THA for patients with high hip dislocation secondary to childhood suppurative arthritis because of aseptic loosening^3^). Complications such as intraoperative femoral fracture (2%) and sciatic nerve paralysis (4.1%) were treated without any sequelae. The major limitations of this study were its retrospective study design, the relatively small number of cases, and the lack of a control group.

### *Conclusion*

This retrospective study showed that the S‐ROM modular femoral stem with subtrochanteric transverse shortening osteotomy, although technically challenging, should be used to treat high hip dislocation secondary to hip pyogenic arthritis because it can provide good results with a low incidence of complications. Significant improvements in clinical function were observed, as were high rates of stable fixation of the cementless implant, restoration of more normal limb lengths, and a low incidence of complications.
